# Human action recognition based on kinematic similarity in real time

**DOI:** 10.1371/journal.pone.0185719

**Published:** 2017-10-26

**Authors:** Qingqiang Wu, Guanghua Xu, Longting Chen, Ailing Luo, Sicong Zhang

**Affiliations:** School of Mechanical Engineering, Xi’an Jiaotong University, Xi’an, China; Universita degli Studi di Verona, ITALY

## Abstract

Human action recognition using 3D pose data has gained a growing interest in the field of computer robotic interfaces and pattern recognition since the availability of hardware to capture human pose. In this paper, we propose a fast, simple, and powerful method of human action recognition based on human kinematic similarity. The key to this method is that the action descriptor consists of joints position, angular velocity and angular acceleration, which can meet the different individual sizes and eliminate the complex normalization. The angular parameters of joints within a short sliding time window (approximately 5 frames) around the current frame are used to express each pose frame of human action sequence. Moreover, three modified KNN (k-nearest-neighbors algorithm) classifiers are employed in our method: one for achieving the confidence of every frame in the training step, one for estimating the frame label of each descriptor, and one for classifying actions. Additional estimating of the frame’s time label makes it possible to address single input frames. This approach can be used on difficult, unsegmented sequences. The proposed method is efficient and can be run in real time. The research shows that many public datasets are irregularly segmented, and a simple method is provided to regularize the datasets. The approach is tested on some challenging datasets such as MSR-Action3D, MSRDailyActivity3D, and UTD-MHAD. The results indicate our method achieves a higher accuracy.

## Introduction

Human action recognition is always an active research topic in recent years [1]. Most previous research on human action recognition are performed on conventional 2D color maps or sequences, many of them based on both global and local spatial-tempo feature [2–4]. Because of the changing of the scale, view angle and lighting condition, it’s hard to deal with the images or sequences correctly. The developing of advanced new depth sensor with low cost such as Kinect makes it possible to use more information to recognize human actions. Compared with traditional RGB data, the depth source can achieve human body silhouettes and get the body posture better [5]. In the past few years, there have been several methods proposed for human action recognition based on color camera and depth sensors. These methods can be roughly divided into two categories by feature source.

In the first category, some researchers took RGB-D images as their feature sources. Many of them used Motion History Images (MHI), Motion Energy Images (MEI) as well as Depth Motion Map (DMM) in different views. In [6], the authors used MHI and selected SVM as their classifier. Yang et al. [7] presented a different method based on MEI, they computed the histogram of oriented gradients from DMM. Similarly, in [8], each depth image was projected into three orthogonal planes, then they employed an L2 regularized collaborative representation classifier for action recognition. Chen et al. [9] also proposed a TriViews framework for this task. They combined the front view, side view and the top view feature descriptors and used Random Forests to classify sequences. In [10], the authors put forward Space-Time Occupancy Patterns (STOP) feature to present actions from depth maps. These methods are mostly proposed in the early stage of human actions recognition field. And their performance are not good enough.

The second category of feature source is based on RGB-D images and skeleton coordinates. Lu Xia and J.K Aggarwal in [11] made use of Local spatio-temporal interest point (STIPs) to describe actions. In [12], the authors built a sample feature model, they used the location velocity and correlation of pose data over time as their descriptor. Mihai Zanfir et al. [13] came up with a Moving Pose (MP) descriptor. The MP consists of position, velocity and acceleration of pose. They used a modified KNN to classify actions, and a complicated normalization step is required in their method. Borghi et al. [14] adopted a set of weighted distributions to model discrete observations for each HMM’s hidden state. Ghorbel et al. [15] also used the position, velocity and acceleration of skeleton as their descriptor. Vemulapalli et al. [16] represented the 3D skeleton as points in a Lie group, then they performed the classification by using a combination of dynamic time warping, Fourier temporal pyramid representation and linear SVM. Ahmad Jalal et al. [17] assumed a HAR framework, they took advantage of detection of human depth silhouettes and joints information. In their description, a Hidden Markov Model is used to classification. Li et al. [18] recognized human action from sequences of depth maps. They employed an action graph and a bag of 3D points from depth images. Yang and Tian in et al. [19] proposed a new feature which combined pose, motion and offset, then they used naïve bayes Nearest Neighbor as their classifier. In [20], Jiang Wang el al. built an actionlet ensemble model to represent each action. They claimed their method was robust to noise. In [21], Yu Zhu et al. combined many descriptors such as Harris 3D detector, histogram of gradient (HOG) and histogram of optical Flow (HOF) in [22], then they used random forests to classify these descriptors. With the developing of deep learning, other researchers tend to choose Neural Network as their classifiers. In Yong Du et al. [23], they proposed a method using normalized skeleton coordinate sequences by Convolution Neural Network (CNN). Vivek Verriah et al. [24] proposed differential Recurrent Neural Network (dRNN) based on Long Short-Term Memory network (LSTM). They claimed that their method can classify any time-series data whatever real-word 2D or 3D human action data. However, most published methods require an entire action sequence in order to get a better classification result. In M. Liu et al. [25], the sequence-based skeletons were transformed into color images. Then they used a multi-stream CNN model to class actions. Mahasseni and Todorovic represented a regularizing LSTM learning algorithm in [26]. Only few techniques that can classify actions cope with the sequence of an action before its end [13,27,28], and not all methods can deal with unsegmented test sequences.

The paper offers several contributions: First, the angular spatio-temporal descriptor is proposed, which not only contains the pose of current frame, but also combines kinematic parameters using a short time window around the current frame to represent actions. Based on the kinematic similarity of humans, the descriptor can reduce the cost of normalization of the skeleton and is more stable. The second contribution is the reduction of the noisy frames in public data. It was determined that many noisy frames exist in action sequences, due to the segmentation of irregular data. The body may remain still during the course of movement, which increases the difficulty of recognition. The proposed method can reduce this effect and achieve a higher accuracy. The final contribution is the computation of the descriptor of each frame and estimation of the time label of the frame. This allows for not only segmented sequences, but also accurate action detection in unsegmented sequences in real time.

The rest of this paper is organized as follows: Section 2 describes the angular spatio-temporal descriptor as well as the method in detail. Section 3 presents the experiment and results on three popular public datasets. Finally, section 4 shows the conclusion and future work.

## Method

### Angular spatio-temporal descriptor

Human actions are often composed of many body movements or poses, such as walking, running, kicking, or jumping. More complex human actions contain interactions with other subjects, for example, drinking water or dancing with others. Although there exists subtle differences between these two types of actions, in this paper, we focus on human actions and movements that are well described by 3D skeleton and without interaction with other bodies.

Because human actions are unpredictable in reality, we assume that the time label (with respect to the staring action frame) of every action frame belonging to a test set is unknown. To training this model, the time label in the training set is known in order. To make ensure the method operates in real time, we have to depend on a few frames to classification quickly. The objective is to obtain as much information as possible from these frames, to ensure robustness.

Actions are usually accompanied by joints rotation motion. Traditional actions recognition methods [1] make use of pose sequences. However, there are many actions for a given pose. For example, pull and push have the same path. However, their direction are different. With the kinematic information of the joints, the pose follows the trend of the next pose. After observing the human motion, we found that whatever the size or sexuality of the body, humans always act in the same angles. The speed and acceleration of angles are steadier than single joint positions for a given action. Many researchers chose the speed of joints and acceleration as the descriptor [11, 14, 15, 17, 24]. Because of the variety of human body’s type, a complex skeleton normalized step is required. A good descriptor should not only include the static pose and the joint kinematics at a given time frame, but also adapt to the different sizes of the human body. Using the speed of joint motion and acceleration to identify the action, the angular velocity and acceleration are used to distinguish the actions sharing similar pose paths but distinct directions.

To acquire angular velocity and acceleration, the velocity and acceleration of every joint must be clear. We assume the motion is composed of a series of continuous joints moving over time. The position of joint {*p*_*i*_(*x*,*y*,*z*),*i* = 1,2…*n*} at time *t* is defined as *P*(*t*), where *n* is the total number of human joints (here *n* = 20). Its second order Taylor expansion in a small time interval around t can be deduced as:

$$P(t)\approx P(t_{0})+P'(t_{0})(t-t_{0})+1/2P''(t_{0}){\left(t-t_{0}\right)}^{2}$$ (1)

For the pose at a given time *t*, it can be expressed by the pose at an earlier time *t*_*0*_ and its first as well as second order derivative. The first and second order derivatives *P*′(*t*) and *P*″(*t*) are deduced using a sliding time window with 5 frames centered at the current frame.

P'(t)=P(t+1)-P(t-1) (2)

P''(t)=P(t+2)+P(t-2)-2P(t) (3)

Even though the three features [*P*(*t*),*P*′(*t*),*P*″(*t*)]present a motion [13], the descriptor is inadequate. Some researchers generalize the descriptor using complex skeleton normalization for each frame. In this method, we employ angle velocity *ω* and angle acceleration *α* to replace *P*′(*t*) and *P*″(*t*).

$$\omega =P'(t)/d_{i}=\frac{P(t+1)\;\text{-}\;P(t\;\text{-}\;1)}{d_{i}}$$ (4)

$$\alpha =P''(t)/d_{i}=\frac{P(t+2)+P(t\;\text{-}\;2)\;\text{-}\;2P(t)}{d_{i}}$$ (5)

The variable *d*_*i*_ is the length of the corresponding skeleton. Finally, the frame descriptor *X*_*t*_ for frame at time label *t* is represented by coordinates of the pose joint and its angle velocity and acceleration over time: X_t_ = [P(t),*λω*,*εα*] = [P(t),*λ*P'(t)/d_*i*_,*ε*P"(t)/d_*i*_]. The parameters *λ* and *ε* are weights of the two derivatives in *X*_*t*_.

To ensure invariability of joint location, the absolute coordinate is converted to the relative coordinate. The hip center joint *p*_*c-hip*_ is chosen as the coordinate origin. The point *p*_*i*_ can also be regarded as a vector. As a result, the point *p*_*i*_ is proportional to *i*-th joint *p*_*c-hip*_, subtracted from the hip joint *p*_*c-i*_ in the absolute coordinate.

$$p_{i}=p_{c-i}-p_{c\;\text{-}\;hip}$$ (6)

We import the proportionality coefficient *η*/*L*_*c–hip*_, where *η* is a constant and *L*_*c–hip*_ is the length of the hip part, as the simple solution to normalize different body types.

$${\vec{p}}_{i}=\frac{\eta }{L_{c-hip}}(p_{c\;\text{-}\;i}-p_{c\;\text{-}\;hip})$$ (7)

At this point, the descriptor *X*_*t*_ is

$$X_{t}=[P(t),\lambda P'(t)/d_{i},\varepsilon P''(t)/d_{i}]=[\frac{\eta }{L_{c-hip}}(p_{c-i}\;\text{-}\;p_{c-hip}),\frac{\lambda }{d_{i}}(p_{c-i}^{t+1}\;\text{-}\;p_{c-i}^{t-1}),\frac{\varepsilon }{d_{i}}(p_{c-i}^{t+2}+p_{c-i}^{t-2}\;\text{-}\;2p_{c-i}^{t})]$$ (8)

When compared to others states of art descriptors, the proposed work has many advantages. Firstly, the descriptor is grounded on raw skeleton coordinates and rapid to obtain. Based on a small time widow around the current frame, the descriptor avoids many calculations. We use a scale factor to fit the different types of the human body, making the normalization uncomplicated without losing robustness. In addition, the introduction of angular velocity and acceleration results in the descriptor having local spatio-temporal information.

### Method overview

**Fig 1:**
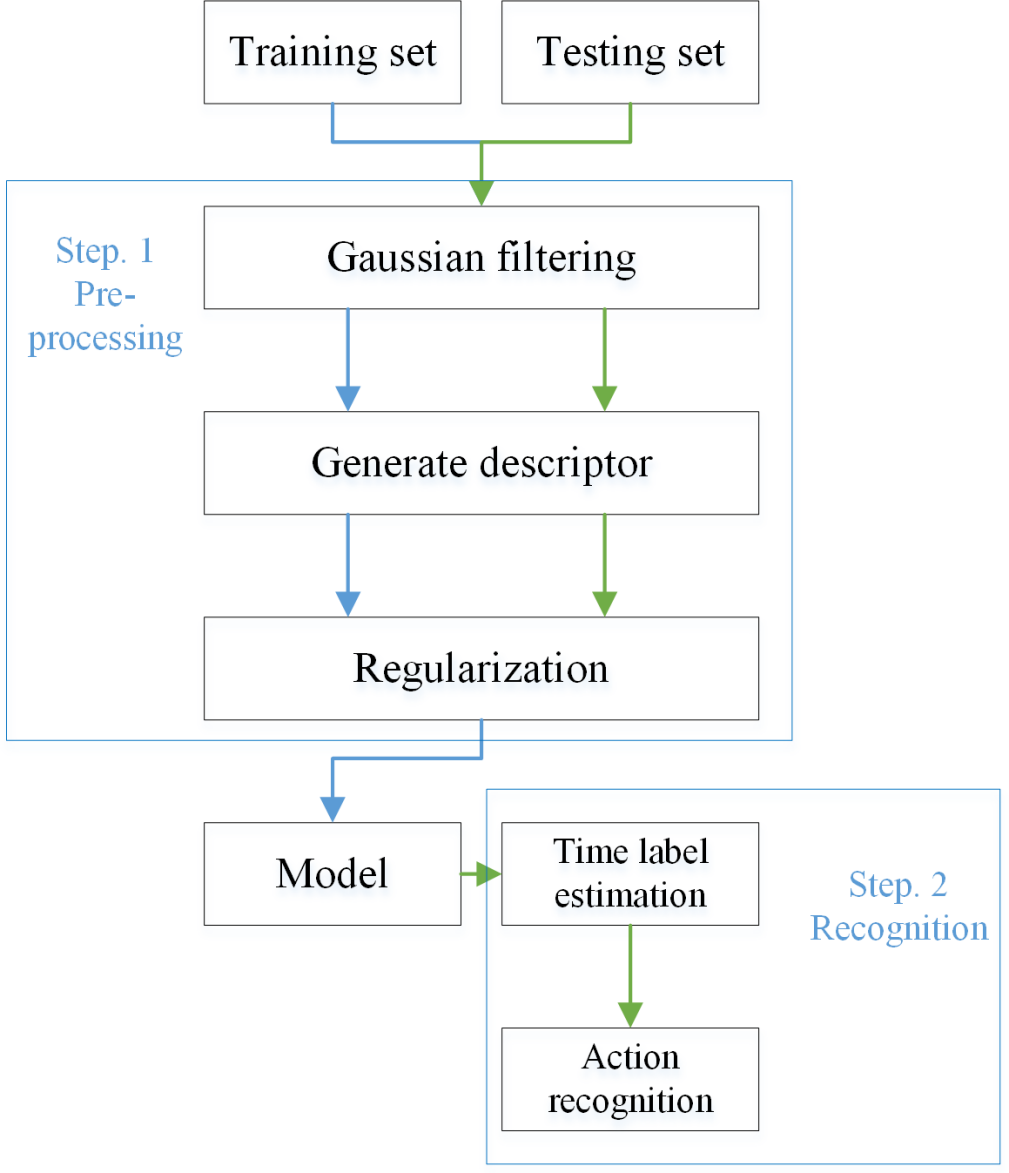
The overview of the suggested approach. The method consists of two stages: Step 1 is the preprocessing of data, and the second step is action recognition. The blue arrow lines show the process of data in training, and the green arrow lines show the recognition of testing data.

The main route of the proposed method was shown in Fig 1. The method can be distributed in two stages. Step 1 is data pre-processing and step 2 is action recognition. The preprocessing includes three parts: Gaussian smoothing, features selection and data regularization. In the action recognition step, the time label of every descriptor was estimated using KNN method firstly. Using the estimated time label and descriptor of input, the action can be classified. A detailed description of this approach is discussed below.

### Pre-processing

#### Gaussian smoothing

Most of the human recognition datasets are based on RGB-D image sensors such as Kinect. Their data may include some noises during the image collection procedure and skeleton estimation. We smooth the action skeleton coordinate using a 5 x 1 Gaussian filter. The standard deviation of the Gaussian filter is 1. Note that the filter can delay 2 frames with respect to the 1st frame.

#### Descriptor generation

Most human body movements includes the head and limbs. To reduce the dimensions of the descriptor vector, we excluded many joints such as the shoulders and neck. The descriptor of each frame can then be computed by the method mentioned above. Our feature includes the local spatio-temporal movement information in a small range.

#### Data regularization

**Fig 2:**
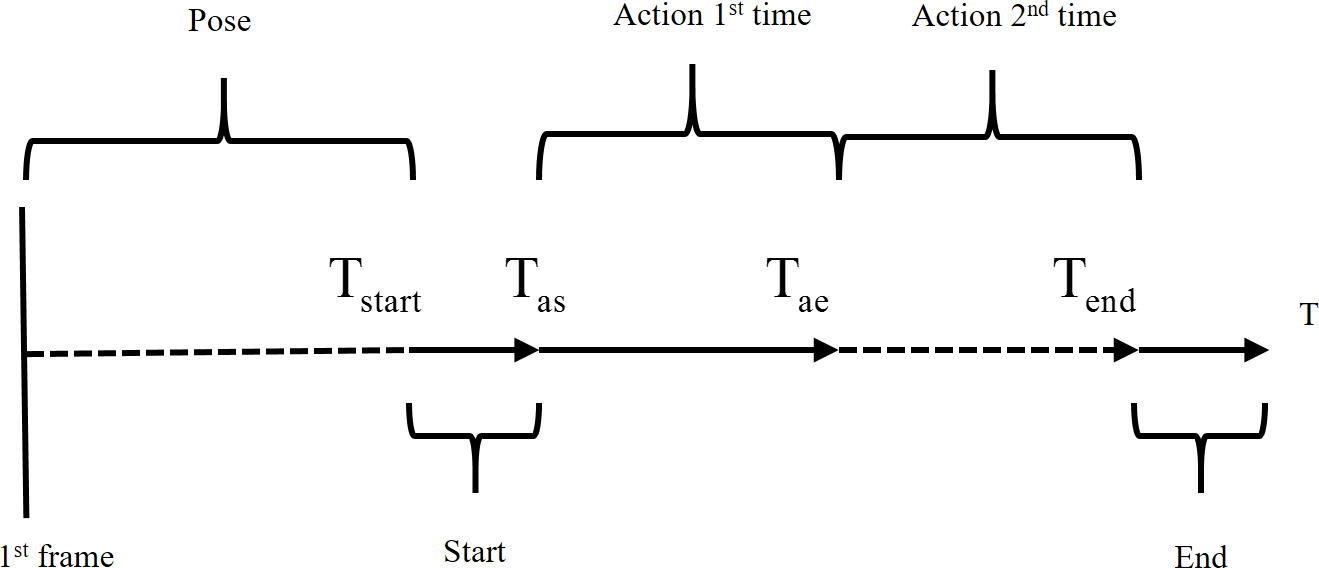
An example of the structure of an action sequence.

Datasets are important for the rapid of development and comparison of algorithms. For better sorting action data, most datasets are segmented in advance according to different actions. Despite this, the segment is not good enough. After observing public action datasets, we found that these datasets have the following characteristics (see Fig 2):

1. The beginning of many action sequences is a fixed posture. That is the first frame is not the true action starting frame. Therefore, it’s hard to locate the true start frame or time *T*_*start*_.
2. Many segment action sequences share the same starting style, which makes distinguishing a few action starting frames difficult. The frames from *T*_*start*_ to *T*_*as*_ are difficult to characterize. They may also have the same end style.
3. Many subjects are performed more than once in an action sequence. This will undoubtedly increase the difficulty of classification in the training.
4. Some of the 3D skeleton coordinates datasets offered are unreliable, especially in occlusion situation.

These adverse factors make action recognition more complex. To increase the accuracy of action classification, we propose the solutions below. First, the true time label of actions must be found. The time window with 5 frames around the current time is employed again. This time, the sliding time window is used to determine if the current frame is active. When human body does not move, the limbs will only shake in a small range due to noise influence. The skeleton coordinates of the limbs and head in the time window [*P*(*t*−2),*P*(*t*−1),*P*(*t*),*P*(*t*+1),*P*(*t*+2)] change little at that moment. The mean square error *S*(*t*) is designed to detect the movement of the limbs and head.

$$S(t)=\frac{1}{m}\sum_{i}^{m}\sqrt{\frac{1}{T}(\sum_{t\;\text{-}\;2}^{t+2}P_{i}^{2})-{\left(\frac{1}{T}\sum_{t\;\text{-}\;2}^{t+2}P_{i}\right)}^{2}}$$ (9)

**Fig 3:**
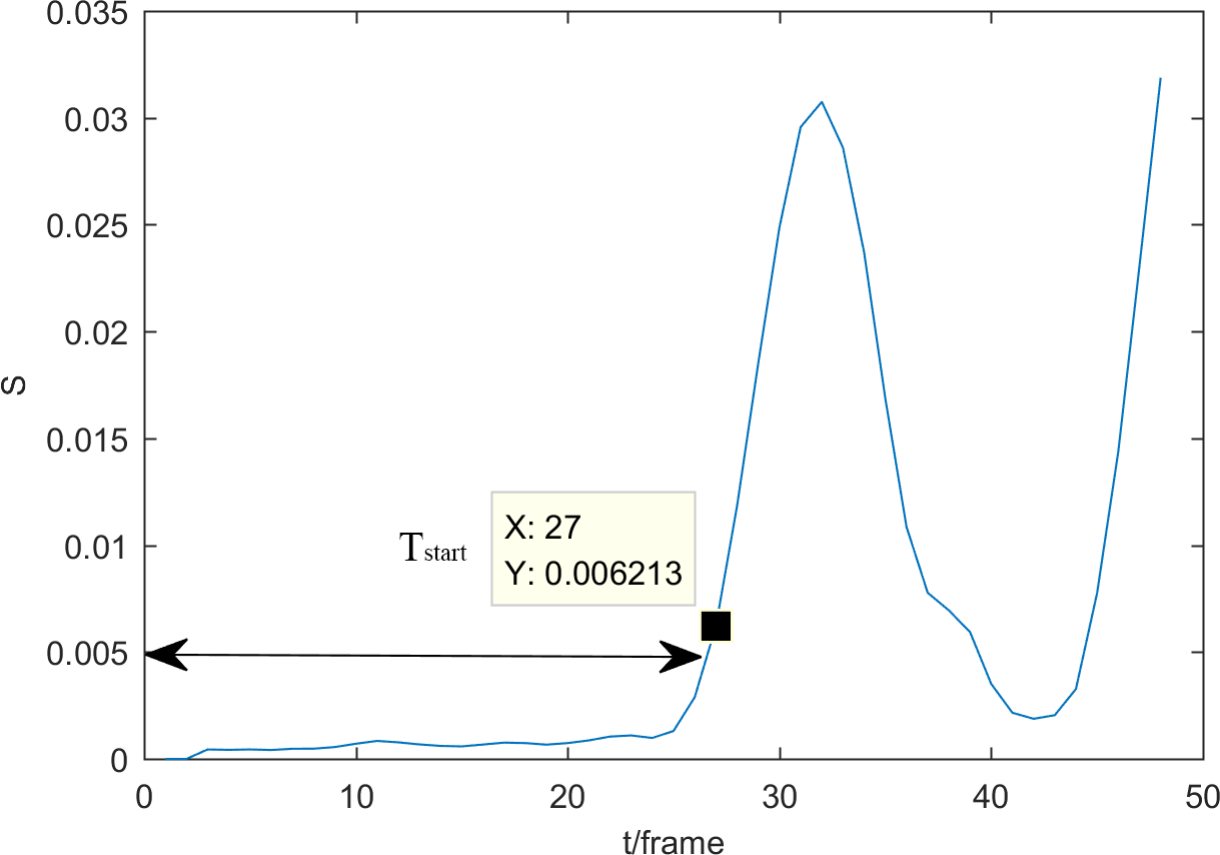
An example of locating the true action start frame. The action ‘high arm wave’ (a01s01e01) in MSR Action 3D was chosen. From frames 1 to 27 (as a result of Gaussian smoothing influence, the sequence number of this frame actually is 30), the subject remains standing. The body starts to raise his hand and has substantial movement.

**Fig 4:**
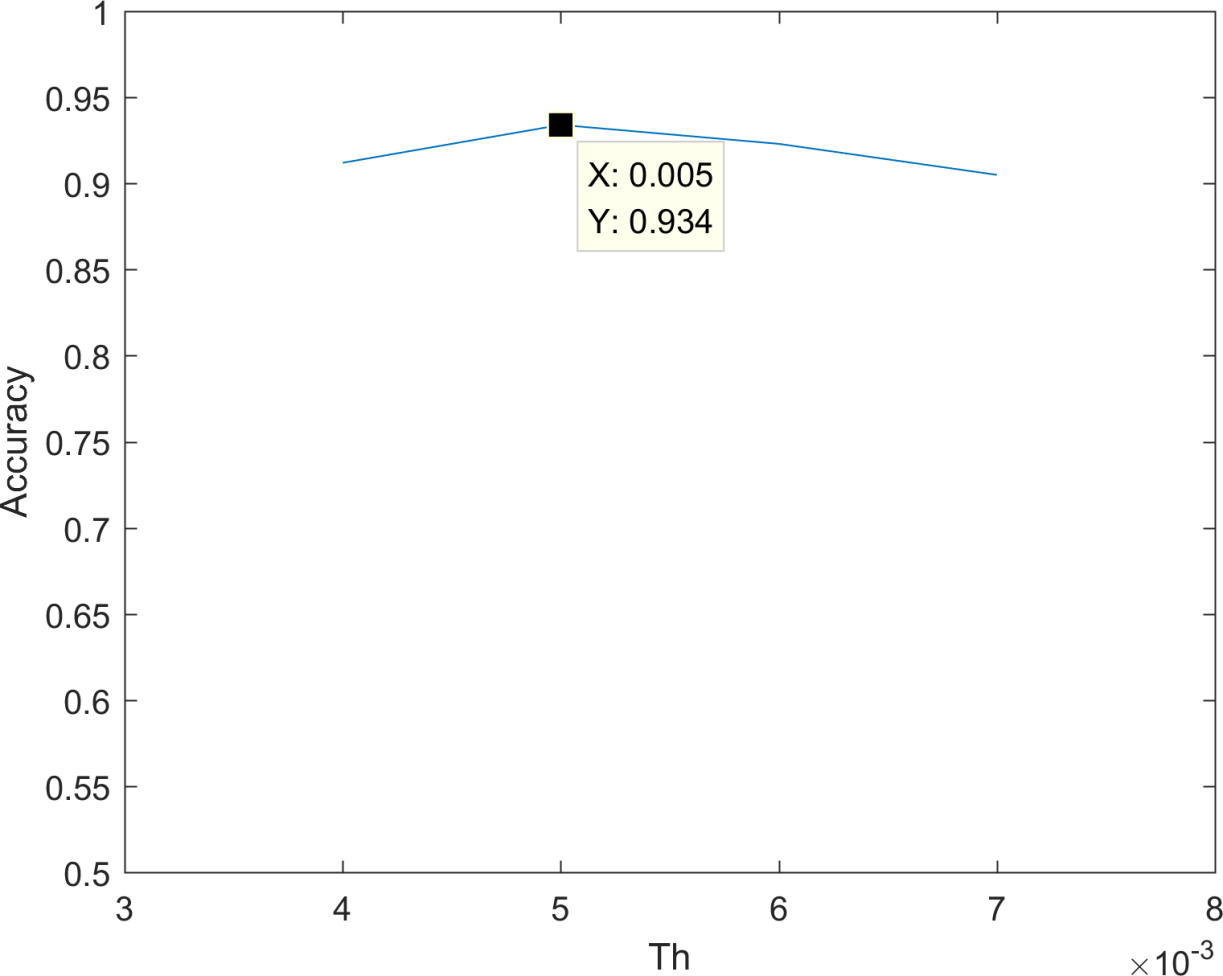
Threshold optimization.

In Eq 9, *T* is the number of frames in the time window (here *T* = 5). Let *m* be the number of joints chosen to detect. *S*(*t*) consists of three values representing the fluctuations of three dimensions. When the minimum of *S*(*t*) is larger than our threshold, the human body is assumed to start moving. We use one action sequence in MSR-Action3D as our example (Fig 3). The purpose of this threshold is to detect the noisy frames. The threshold is approximately 0.005, according to experience gained in the MSRAction3D dataset. To optimize the threshold, we chose the threshold *Th* = [0.004,0.005,0.006,0.007], with the result is shown in Fig 4. When *Th* = 0.005, the accuracy is the highest.

We use a confidence parameter of every frame in the sequence based on KNN algorithm, to solve the second challenge. We design those that are common to other actions having a smaller confidence of the current action.

Although the descriptor already fused local space and time information, it’s short of the global temporal ordering. The global time location of the descriptor in the entire action sequence is often significant. For example, when a subject does the “draw tick” action, he usually moves his hand downwards, then moves it to obliquely above. The order of these two movement matters. What’s more, the second movement is the biggest difference from the path of “hammer”. To fuse global time information, the solution chooses the samples that are located at a similar time label with, respect to the true first frame *T*_*start*_ (estimated above) in the training sequences as the neighbors to vote. By integrating the descriptor that has already fused local time and space information with the global temporal time window, the modified KNN can account for its shortage. Furthermore, the method processes single input descriptor in real time.

To estimate the confidence of every training frame, we consider the action label of every frame in training set as unknown, and use the modified KNN method to classify the frame using the other training frames. For a training frame descriptor *X*_*t*_ belonging to action A, there are *K*_*t*_ samples that are also classified to action A, close to the *X*_*t*_, with *K* votes in total. The confidence of the current frame *c*(*X*_*t*_) can be approximated by Eq 10.

$$c(X_{t})=w(A)\frac{K_{t}}{K}$$ (10)

Since the duration of every sequence differs greatly, w(A) is the weight of different action sequences to balance.

### Action recognition

Traditional human action recognition methods focused on segmented action sequences. This limits them to classifying the actions in real time. In this method, the input of the classifier is a series of descriptors based on just five frames around the current time label. The before and after time label of input frames are independent, considering single input each time as well as the continuity of different actions in reality. The goal of this work is to discern the action sequences using a certain amount of frames rather than the entire sequence. We aim to recognize the continuous unsegmented actions in reality. This means the time label of every frame is unknown, allowing the input frame descriptor to be the changing frame of two consecutive different actions. This means the time label must be estimated before action recognition.

#### Estimate frame

The speed of identical actions is assumed to be similar among all subjects in this method. Once the start time labels of the same actions are aligned, the angular velocity of joints will be similar at the same frame label.

One of the difficulties mentioned above in public action datasets is that many subjects are performed more than once in a single action sequence. The time label of the duplicate action frames is wrong if they only use timing sequence information. As a result, the time label is an important factor in this method, to avoid mistake of time label deduction. For example, in many methods, if the current frame is *t*_*1*_, the subsequent frame would be treated as *t*_*1*_ + 1. However, the subsequent frame also has the possibility of belonging to the second time action or another action. The estimation step provides another benefit in estimating the time label of frames, even if they are belong to two consecutive different actions. This type of movement is very common in reality.

In our estimated frame procedure, a standard KNN classifier estimates the time label of every input descriptor. For an input descriptor in the testing set, all samples in the training set vote for this input descriptor. The frame labels in training set are also calibrated. In Eq 11, *T*_*i*_ is the estimated frame label of the input frame, and *v*(*X*) represent the count of votes belong to action *X*. In our test, most of the estimated frame labels are close to the true regularized frame labels in the testing set. While different actions may have overlapping parts, leading to the incorrect estimation, the recognition part is based on many input frames. Therefore, the impact of a few wrong estimations on action recognition is very small. It also indirectly indicates that the overlapping parts of frames are not the key frames used to present actions.

$$T_{i}=\arg\;\max\sum v(X)$$ (11)

#### Recognition

**Fig 5:**
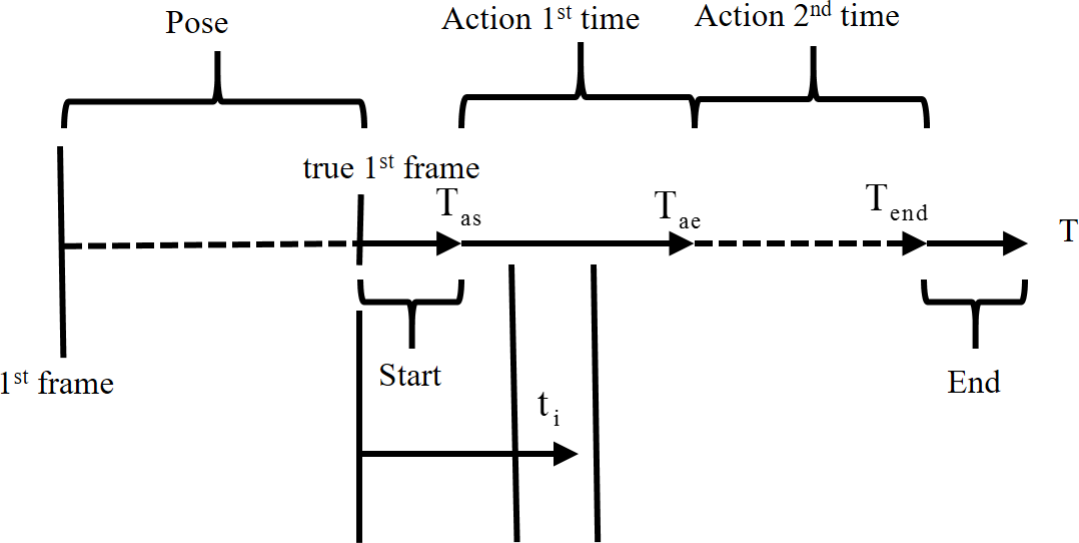
Frames in the range of the time window near *t*_*i*_ can vote for classification.

The proposed angular spatial-temporal descriptor includes the kinematic information of joints to describe action sequences. An action classifier was built using the modified KNN method previously mentioned. In this time, the weight of vote in the modified KNN is replaced by the confidence of the nearest frames, *c(X)*. The general route of this approach is as follows. At the estimated time label *t*_*i*_, the frames that are in a time window of the frames around *t*_*i*_ in the training set vote for classification (see Fig 5). There are *kt* votes from the first frame to *t* in total. When enough frames were observed (the threshold frame label is set as *T*_*th*_), the votes of every action arecounted, and the most supported class is determined that meets the condition:

$$\frac{\max(\sum_{t_{i}}^{T}c(X_{i}))}{\sum_{a}^{}\sum_{t_{i}}^{T}c(X_{i})}>\alpha \;(T>T_{th}),$$

where *α* is a constant threshold. This guarantees the sufficient frames in the action ***A*** can be correctly classified. The input action sequence is action ***A***, determined by the following equation.

$$A=\arg\;\max\sum_{t_{i}}^{T}c(X_{i})$$ (12)

This methods takes advantage of the probability of coordinates offered to improve the accuracy.

## Experiment & results

To verify the proposed method, we test our approach using public action datasets. We use MSR-Action3D, MSRDailyActivity3D and UTD-MHAD, and compare the result to other state-of-the art methods using these datasets. This also reveals the necessity of the pre-processing step in the training set and the reliability of time label estimation. This method is executed on a computer with win10 system, 3.2GHz CPU and 4GB RAM, using MATLAB version R2014a. The method precisely recognizes actions in real-time speeds of 250–500 FPS with approximately 2 frames delay.

### MSR Action3D

The MSR Action3D dataset was built by Microsoft, and the action sequences were captured by a RGB-D camera. The dataset consists of approximately 20 actions, each action performed 2 or 3 times by 10 subjects. There are 567 sequences in total with 10 sequences were not used due to [18] missing data. The dataset offers 3D skeleton joint coordinates and precision of coordinates.

#### Segmented dataset

**Fig 6:**
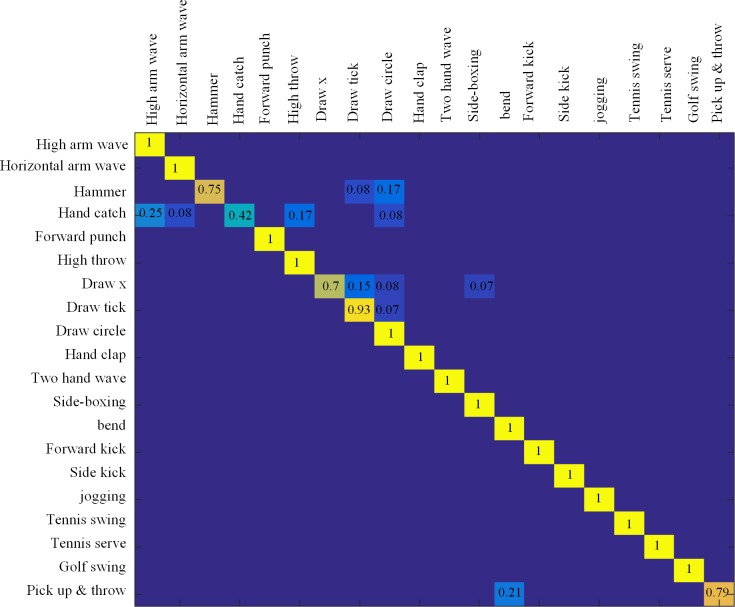
The confusion matrix on the MSR Action 3D dataset with segmented sequence. A total of 15/20 actions are perfectly classified.

First, the dataset is regarded as the segmented data. That is, every frame in the sequence is known in advance. We use the cross-subject test setting with subjects (2,3,5,7,9) as training set and subjects (1,4,6,8,10) as testing set. The parameters *λ* and *ε* in *X*_*t*_ were optimized, and remained constant over all tests (*λ* = 0.8, *ε* = 0.6). Fig 6 shows the confusion matrix result on the MSR Action 3D dataset under the segmented condition. There were 15 out of 20 actions that were perfect classified. The average accuracy of our test was 93.4%. Table 1 shows the comparison with current methods on the MSR Action 3D dataset. Compared to [17], the results (Table 1) show that this method improves the accuracy of the current state of the technology by 1%. Their average accuracy was 92.4%, and they used leave one-sub out cross test to evaluate their results, meaning, more subjects were used for training in each round. In spite of 17 of 20 actions being perfectly classified in [13], they have the fatal recognition error in the ‘hammer’ and ‘hand catch’. However the accuracy of our method in these two actions still occupies the largest proportion. Despite this, the accuracy of our approach is higher than [13].

**Table 1:** Comparison of recognition accuracy of proposed method on MSR Action 3D.

Method	Accuracy (%)
Actionlet ensemble [20]	88.2
Lie group [16]	89.48
Moving Pose[13]	91.7
2-order dRNN [24]	92.03
HMM method [17]	92.4
Proposed method	93.4

#### Unsegmented dataset

Since this was an unsegmented case, the length of actions performed is unknown, as well as the time label of each frame. The only difference between unsegmented and segmented data is that the timing of each frame as input is unknown in the unsegmented data. We use the cross-subject test setting with subjects (2,3,5,7,9) as the training set and the reaminings as testing set. In this method, every frame in the test sequences as treated as independent. To test the efficient time label estimation, every descriptor was estimated its time label by using KNN. Then the recognition step was then performed.

**Fig 7:**
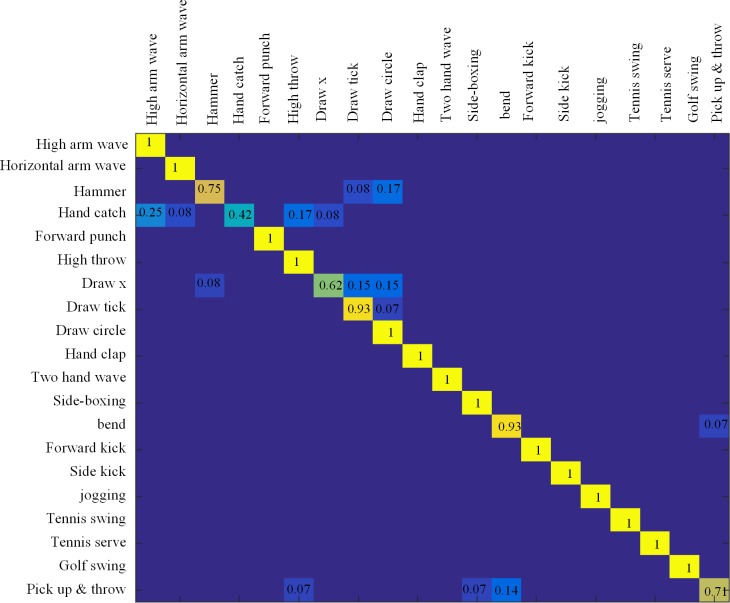
The confusion matrix on MSR Action 3D dataset with unsegmented sequence. Average accuracy: 92.34%.

The result is shown in Fig 7, and the accuracy of the MSR Action 3D dataset on the unsegmented condition is 92.34%, lower than segment condition by 1%. That is because the front part of the action ‘golfing and throw’ is similar to the latter part of the action ‘high throw’, and the coordinates of the bend part in action 20 are incorrectly identified, which increases the difficulty of recognition. Even though our method performs well on the dataset, it also demonstrates that the estimation step in the action classification is effectual. Table 2 shows the comparison of the moving pose method [13] with ours on unsegmented action detection. In [13], a sliding window approach was used to estimate the probability of actions through KNN in the window. And their accuracy in MSR Action3D is about 0.89. The accuracy of ours is higher than their by 0.3.

**Table 2:** Unsegmented action detection performance on MSR-Action3D.

Methods	Accuracy (%)
Moving pose [13]	89
Proposed method	92.3

### MSR-Daily Activity3D

The MSRDailyActivity3D dataset contains 16 activities performed by 10 subjects. Each activity was performed twice. The dataset consists of 320 videos with RGB, depth and joints in total. The dataset is challenging, because many activities are performed in a similar fashion. What’s more, the subject may remain in one pose for an indefinite time. The subjects (1,3,5,7,9) were used as training set, while the others were used as testing set (*λ* = 1.2, *ε* = 0.6). The cross-subject test was also used on the dataset. We consider the dataset in the unsegmented case, which means the estimation step is required. Table 3 shows the comparison of many current methods based on skeletons.

**Table 3:** Comparison of recognition accuracy of proposed method on MSRDailyActivity 3D.

Methods	Accuracy (%)
Moving pose [13]	73.8
Learning Dictionaries [29]	79.3
Ours	76.9

Our accuracy on MSRDailyActivity3D dataset under unsegmented condition is 76.9%. The accuracy of our method is lower than [29]. This is beacuse they divided the dataset into 3 subsets. This undoubtedly reduced the difficulty of the classification. We found that the time for different subjects to remain in a pose in action is very different. For example, the length of the drinking pose in different sequences differs greatly. The regularization of training sets can reduce the gap.

### UTD-MHAD

UTD-MHAD [30] is a multimodal action dataset, captured by one Microsoft Kinect camera and one wearable inertial sensor. The dataset contains 27 actions performed by 8 subjects (4 females and 4 males) with each subject performing each action 4 times. Cross-subjects protocol is adopted as in [30] on this dataset. The data from the subject numbers 1, 3, 5, 7 were used for training and subject numbers 2, 4, 6, 8 were used for testing. Table 4 shows the comparison of the performance of the proposed method and that reported in [30,31]. Note that the method used in [30] is based on depth and inertial sensor data, not skeleton data alone. (*λ* = 0.8, *ε* = 0.6)

**Table 4:** Comparison of recognition accuracy of proposed method on UTD-MHAD.

Method	Accuracy (%)
Kinect & Inertial [30]	79.1
CNN [31]	84.81
ours	90.47

**Fig 8:**
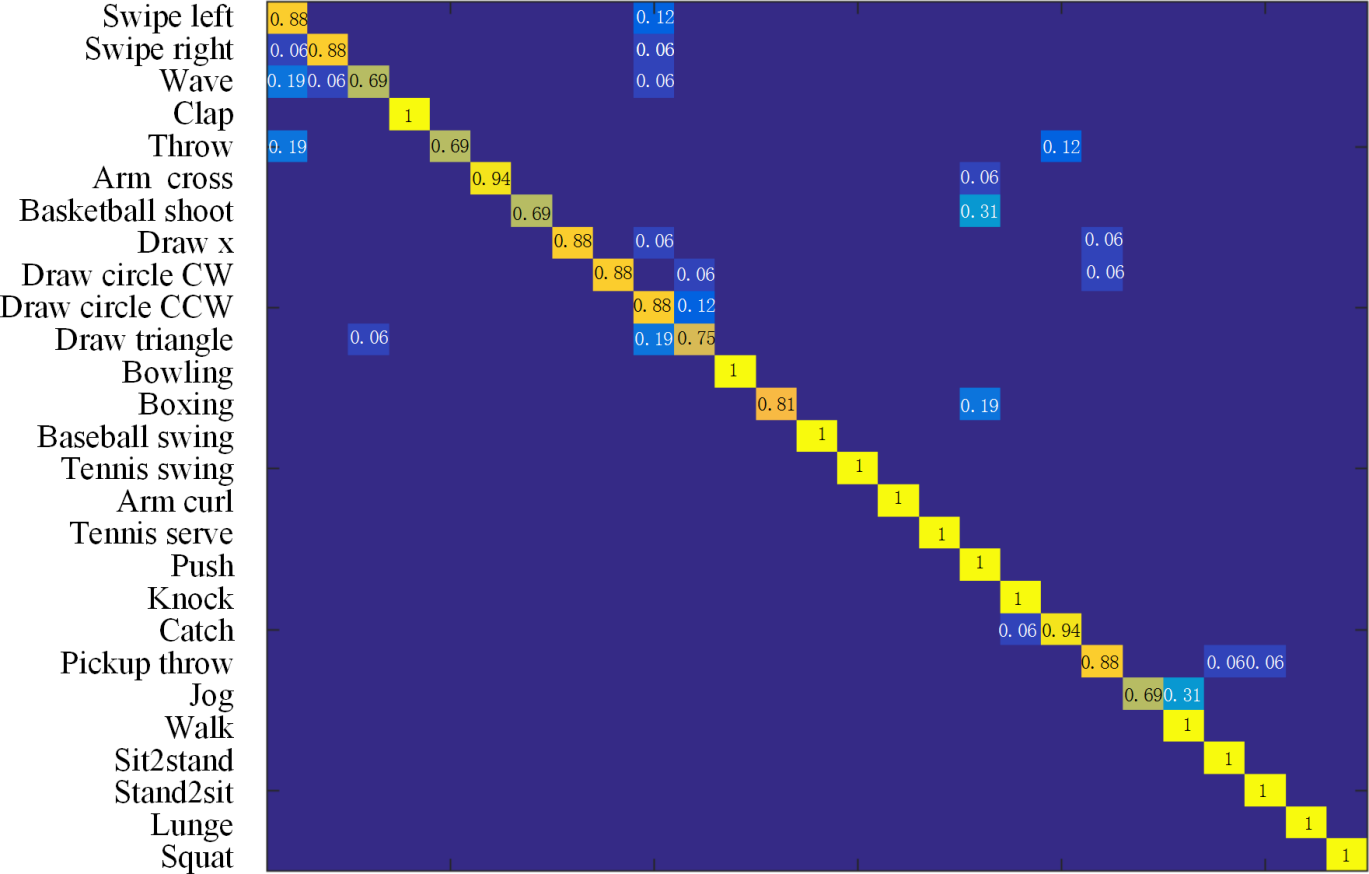
The confusion matrix on MSR Action 3D dataset on UTD-MHAD. Average accuracy: 90.47%.

The confusion matrix is shown in Fig 8. Compared to the previous two datasets, this dataset is much more challenging. From the confusion matrix, the proposed method cannot distinguish some actions well, such as, “Draw circle CCW" and “Draw triangle". A probable reason is that the paths of the two actions are similar. And have the same direction of rotation.

## Conclusion

We proposed a novel angular spatio-temporal descriptor for human action recognition. We also presented a preprocessing method based on statistics to detect every stage of actions. We consider each frame in sequence as independent and put forward a classifier to estimate each frame’s time label, making the action recognition in real time. We showed the superiority of our method, compared to others state-of-the-art, regardless of the dataset being segmented or unsegmented. Due to our method being based on the assumption that the speed of human motion is similar, this work didn’t consider the disabled or elderly. In future work, we plan to explore the action recognition for these two populations to allow for better communication with computers in HCI.

## References

[1] Zhang J, Li W, Ogunbona PO, Wang P, Tang C. RGB-D-based action recognition datasets: A survey. Pattern Recognition. 2016;60:86–105.

[2] Poppe R. A survey on vision-based human action recognition. Image and vision computing. 2010;28(6):976–90.

[3] Aggarwal JK, Ryoo MS. Human activity analysis: A review. ACM Computing Surveys (CSUR). 2011;43(3):16.

[4] Moeslund TB, Hilton A, Krüger V. A survey of advances in vision-based human motion capture and analysis. Computer vision and image understanding. 2006;104(2):90–126.

[5] Shotton J, Sharp T, Kipman A, Fitzgibbon A, Finocchio M, Blake A, et al Real-time human pose recognition in parts from single depth images. Communications of the ACM. 2013;56(1):116–24.

[6] Megavannan V, Agarwal B, Babu RV, editors. Human action recognition using depth maps. Signal Processing and Communications (SPCOM), 2012 International Conference on; 2012: IEEE.

[7] Yang X, Zhang C, Tian Y, editors. Recognizing actions using depth motion maps-based histograms of oriented gradients. Proceedings of the 20th ACM international conference on Multimedia; 2012: ACM.

[8] Chen C, Liu K, Kehtarnavaz N. Real-time human action recognition based on depth motion maps. Journal of real-time image processing. 2016;12(1):155–63.

[9] Chen W, Guo G. TriViews: A general framework to use 3D depth data effectively for action recognition. Journal of Visual Communication and Image Representation. 2015;26:182–91.

[10] Vieira A, Nascimento E, Oliveira G, Liu Z, Campos M. Stop: Space-time occupancy patterns for 3d action recognition from depth map sequences. Progress in Pattern Recognition, Image Analysis, Computer Vision, and Applications. 2012:252–9.

[11] Xia L, Aggarwal JK (2013) Spatio-temporal Depth Cuboid Similarity Feature for Activity Recognition Using Depth Camera. 9: 2834–2841.

[12] Eweiwi A, Cheema MS, Bauckhage C, Gall J, editors. Efficient pose-based action recognition. Asian Conference on Computer Vision; 2014: Springer.

[13] Zanfir M, Leordeanu M, Sminchisescu C, editors. The moving pose: An efficient 3d kinematics descriptor for low-latency action recognition and detection. Proceedings of the IEEE International Conference on Computer Vision; 2013.

[14] Borghi G, Vezzani R, Cucchiara R. Fast gesture recognition with Multiple Stream Discrete HMMs on 3D skeletons; 2016. IEEE. pp. 997–1002.

[15] Ghorbel E, Boutteau R, Bonnaert J, Savatier X, Lecoeuche S. A fast and accurate motion descriptor for human action recognition applications; 2016. IEEE. pp. 919–924.

[16] Vemulapalli R, Arrate F, Chellappa R. Human action recognition by representing 3d skeletons as points in a lie group; 2014. pp. 588–595.

[17] Jalal A, Kamal S, Kim D. Human Depth Sensors-Based Activity Recognition Using Spatiotemporal Features and Hidden Markov Model for Smart Environments. Journal of Computer Networks and Communications. 2016;2016:5.

[18] Li W, Zhang Z, Liu Z, editors. Action recognition based on a bag of 3d points. Computer Vision and Pattern Recognition Workshops (CVPRW), 2010 IEEE Computer Society Conference on; 2010: IEEE.

[19] Yang X, Tian YL, editors. Eigenjoints-based action recognition using naive-bayes-nearest-neighbor. Computer vision and pattern recognition workshops (CVPRW), 2012 IEEE computer society conference on; 2012: IEEE.

[20] Wang J, Liu Z, Wu Y, Yuan J, editors. Mining actionlet ensemble for action recognition with depth cameras. Computer Vision and Pattern Recognition (CVPR), 2012 IEEE Conference on; 2012: IEEE.

[21] Zhu Y, Chen W, Guo G, editors. Fusing spatiotemporal features and joints for 3d action recognition. Proceedings of the IEEE Conference on Computer Vision and Pattern Recognition Workshops; 2013.

[22] Laptev I, Marszalek M, Schmid C, Rozenfeld B, editors. Learning realistic human actions from movies. Computer Vision and Pattern Recognition, 2008 CVPR 2008 IEEE Conference on; 2008: IEEE.

[23] Du Y, Fu Y, Wang L, editors. Skeleton based action recognition with convolutional neural network. Pattern Recognition (ACPR), 2015 3rd IAPR Asian Conference on; 2015: IEEE.

[24] Veeriah V, Zhuang N, Qi G-J, editors. Differential recurrent neural networks for action recognition. Proceedings of the IEEE International Conference on Computer Vision; 2015.

[25] Liu M, Liu H, Chen C (2017) Enhanced skeleton visualization for view invariant human action recognition. Pattern Recognition 68: 346–362.

[26] Mahasseni B, Todorovic S. Regularizing long short term memory with 3D human-skeleton sequences for action recognition; 2016. pp. 3054–3062.

[27] Ellis C, Masood SZ, Tappen MF, Laviola JJ, Sukthankar R (2013) Exploring the Trade-off Between Accuracy and Observational Latency in Action Recognition. International Journal of Computer Vision 101: 420–436.

[28] Hoai M, Torre FDL, editors. Max-margin early event detectors. Computer Vision and Pattern Recognition; 2014.

[29] Yang Z. Learning dictionaries of sparse codes of 3D movements of body joints for real-time human activity understanding. Plos One. 2014;9(12):e114147 doi: 10.1371/journal.pone.0114147 25473850 10.1371/journal.pone.0114147 PMC4256388

[30] Chen C, Jafari R, Kehtarnavaz N. Utd-mhad: A multimodal dataset for human action recognition utilizing a depth camera and a wearable inertial sensor; 2015. IEEE. pp. 168-172.Qi J,

[31] Wang P, Li Z, Hou Y, Li W. Action recognition based on joint trajectory maps using convolutional neural networks; 2016. ACM. pp. 102–106.

